# From policy to practice: exploring the implementation of antiretroviral therapy access and retention policies between 2013 and 2016 in six sub-Saharan African countries

**DOI:** 10.1186/s12913-017-2678-1

**Published:** 2017-11-21

**Authors:** Julie Ambia, Jenny Renju, Alison Wringe, Jim Todd, Eveline Geubbels, Jessica Nakiyingi-Miiro, Mark Urassa, Tom Lutalo, Amelia C. Crampin, Daniel Kwaro, Catherine Kyobutungi, Natsayi Chimbindi, F. Xavier Gomez-Olive, Malebogo Tlhajoane, Brian Njamwea, Basia Zaba, Paul Mee

**Affiliations:** 1 London School of Hygiene and Tropical Medicine, London, UK; 2 Ifakara Health Institute, Dar-es-Salaam, Tanzania; 3 Medical Research Council/Uganda Virus Research Institute, Entebbe, Uganda; 4 National Institute for Medical Research, Mwanza, Tanzania; 5 Rakai Health Sciences Program, Kampala, Uganda; 6 Malawi Epidemiology and Intervention Research Unit, Lilongwe, Malawi; 7 Kenya Medical Research Institute/Centers for Disease Control and Prevention, Kisumu, Kenya; 8 Africa Population Health Research Center, Nairobi, Kenya; 9 Africa Health Research Institute, Durban, South Africa; 10 MRC/Wits Rural Public Health and Health Transitions Research Unit, University of the Witwatersrand, Johannesburg, South Africa; 11 Imperial College, London, United Kingdom, and Biomedical Research and Training Institute, Harare, Zimbabwe

**Keywords:** WHO guidelines, Policy review, Health facility survey, ART, Retention, Access, Africa, HIV, AIDS

## Abstract

**Background:**

Understanding the implementation of 2013 World Health Organization (WHO) consolidated guidelines on the use of antiretroviral drugs for treating and preventing HIV infection at the facility level provides important lessons for the roll-out of future HIV policies.

**Methods:**

A national policy review was conducted in six sub-Saharan African countries to map the inclusion of the 2013 WHO HIV treatment recommendations. Twenty indicators of policy adoption were selected to measure ART access (*n* = 12) and retention (*n* = 8). Two sequential cross-sectional surveys were conducted in facilities between 2013/2015 (round 1) and 2015/2016 (round 2) from ten health and demographic surveillance sites in Kenya, Malawi, South Africa, Tanzania, Uganda and Zimbabwe. Using standardised questionnaires, facility managers were interviewed. Descriptive analyses were used to assess the change in the proportion of facilities that implemented these policy indicators between rounds.

**Results:**

Although, expansion of ART access was explicitly stated in all countries’ policies, most lacked policies that enhanced retention. Overall, 145 facilities were included in both rounds. The proportion of facilities that initiated ART at CD4 counts of 500 or less cells/μL increased between round 1 and 2 from 12 to 68%, and facilities initiating patients on 2013 WHO recommended ART regimen increased from 42 to 87%. There were no changes in the proportion of facilities reporting stock-outs of first-line ART in the past year (18 to 11%) nor in the provision of three-month supply of ART (43 to 38%). None of the facilities provided community-based ART delivery.

**Conclusion:**

The increase in ART initiation CD4 threshold in most countries, and substantial improvements made in the provision of WHO recommended first-line ART regimens demonstrates that rapid adoption of WHO recommendations is possible. However, improved logistics and resources and/or changes in policy are required to further minimise ART stock-outs and allow lay cadres to dispense ART in the community. Increased efforts are needed to offer longer durations between clinic visits, a strategy purported to improve retention. These changes will be important as countries move to implement the revised 2015 WHO guidelines to initiate all HIV positive people onto ART regardless of their immune status.

**Electronic supplementary material:**

The online version of this article (10.1186/s12913-017-2678-1) contains supplementary material, which is available to authorized users.

## Background

In 2015, 10.3 million people were receiving antiretroviral therapy (ART) in Eastern and Southern Africa compared with 4 million in 2010, accounting for 60% of the people receiving ART globally [1]. This number is expected to increase further following revisions to the ART eligibility criteria in the 2015 World Health Organisation (WHO) guidelines that recommend treatment initiation for everyone who is HIV-infected, regardless of their immunological status (dubbed “universal test and treat”) [2]. Implementing these new guidelines will be an essential step towards achieving the UNAIDS “90–90-90” target (i.e. by 2020, 90% of HIV-infected people will be diagnosed, 90% of the individuals diagnosed will receive sustained ART and 90% of the individuals on ART will achieve viral suppression) in order to eliminate AIDS by 2030 [1].

A recent systematic review of studies published between 2008 and 2013 reported that almost a quarter of adult patients in sub-Saharan Africa (SSA) were lost to follow-up (LTFU) within one year of starting ART [3] clearly challenging attainment of these ambitious targets. Various health systems factors have been cited as barriers to retention in care including repeated stock-outs of ART, lack of on-site CD4 testing, prolonged delays before receiving viral load results, and long waiting times for consultations [4, 5]. The 2015 WHO guidance included different approaches intended to improve access and retention in HIV care and treatment programmes. However the rate at which countries adopt and subsequently implement these guidelines varies. For instance, it took an average of 24 months for most countries to adopt the 2010 WHO ART guidelines [6]. Between December 2013 and May 2015, Kenya, Uganda, Tanzania, Malawi, Zimbabwe and South Africa revised their national ART guidelines to make them consistent with the 2013 WHO guidelines on treatment eligibility [7]. In these countries, the time-period between publication of the 2013 WHO guidelines on treatment eligibility (i.e. June 2013) and the revision of national ART guidelines averaged 12 months (range 6–23 months).

Understanding the implementation of the WHO 2013 guidance on the use of antiretroviral drugs for treating and preventing HIV infection at the health facility level will help to identify existing gaps in HIV service provision. Addressing these gaps is likely to be an important step in ensuring health systems readiness for the implementation of future HIV policies including those on universal “test and treat”. In this study, we assess the adoption of the 2013 WHO HIV treatment and prevention recommendations into national guidelines in six African countries between 2013 and 2016. We additionally describe progress in the implementation of national policies on ART access and retention within the health facilities serving the populations of rural health and demographic surveillance sites (HDSS) in each country over the same time-period.

## Methods

### Policy review

We updated a previous policy review conducted by Church et al., [8] in which national HIV policies published between January 2003 and June 2013 were documented in six sub-Saharan African countries. The focus of the updated policy review was to assess the uptake of the 2013 WHO recommendations related to the eligibility threshold for ART initiation, the availability of first-line ART regimes, and recommendations to improve retention. Internet searches were conducted to obtain national guidelines, addendums to the national ART guidelines, and clinical guidelines for the management of ART patients published between July 2013 and July 2015.

### Health facility survey

**Fig. 1:**
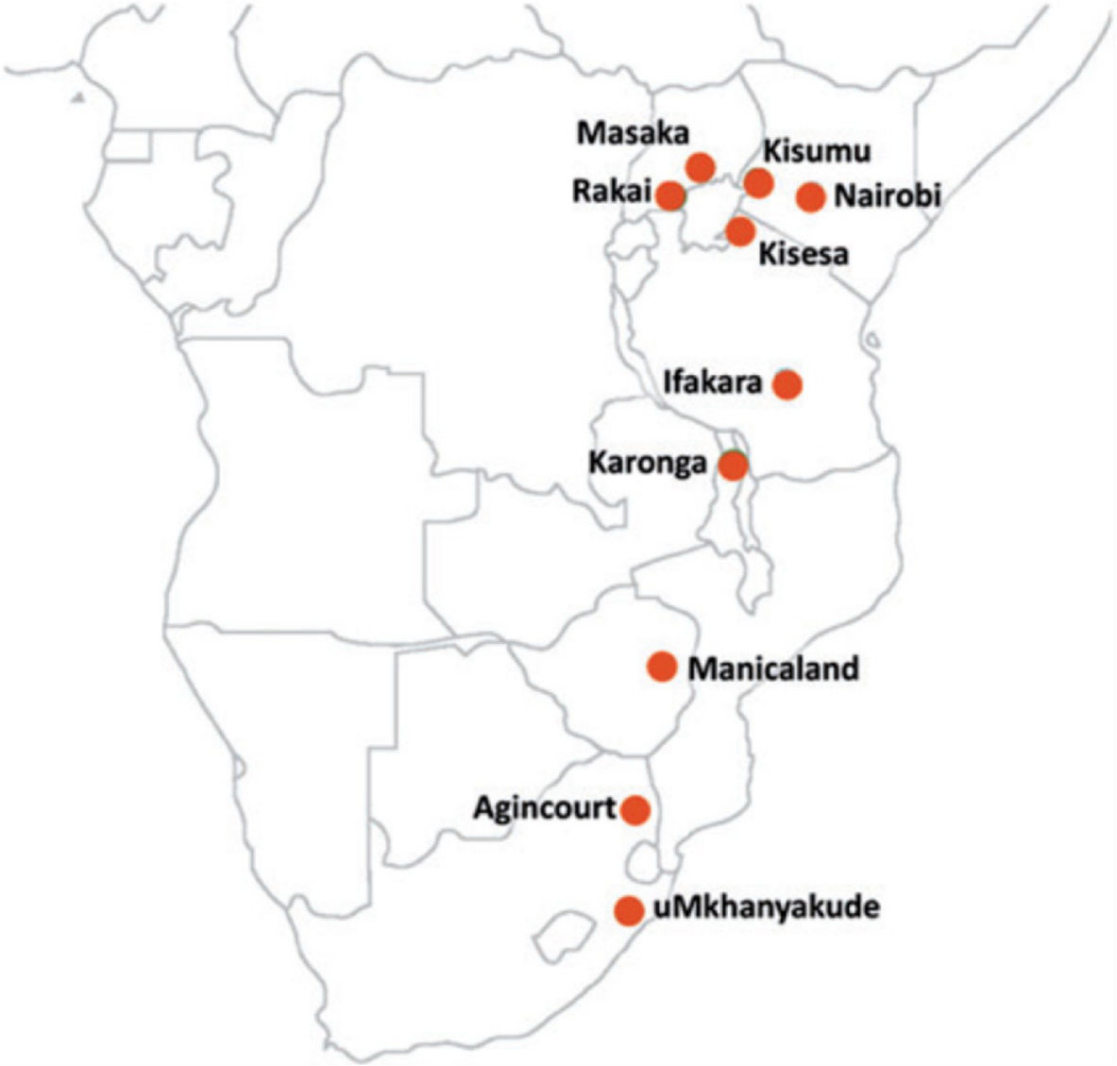
Location of the health and demographic surveillance systems

We surveyed health facilities located within or on the border of ten HDSS in Kenya (Nairobi and Kisumu), Malawi (Karonga), South Africa (Agincourt and uMkhanyakude), Tanzania (Ifakara and Kisesa), Uganda (Masaka and Rakai) and Zimbabwe (Manicaland) (Fig. 1). These HDSS are part of the network for Analysing Population-based HIV/AIDS data on Africa (ALPHA) [9] and conduct population-based HIV surveillance in eastern and southern Africa (http://alpha.lshtm.ac.uk/). All facilities serving the population of the HDSS sites were sampled except in Nairobi, where a convenience sample was selected and in Ifakara, where only facilities with more than 100 ART patients were included.

Cross-sectional health facility surveys were conducted between 2013 and 2015 (mostly 2013–2014) (round 1) and between 2015 and 2016 (round 2). At the time of round 1, all countries were using the 2010 WHO HIV prevention and treatment guidelines on ART eligibility, except Uganda where the 2013 WHO guidelines were already in place. By round 2, all countries had adopted the 2013 WHO guidelines.

Face-to-face interviews were conducted in English with the staff manager (in small facilities) or the person managing ART services (in large facilities) using a structured questionnaire. The design of the questionnaire has been described elsewhere [10]. Briefly, a conceptual framework that included five thematic areas and 54 corresponding indicators derived from a systematic review of literature, WHO guidelines and expert reviews informed the content of the questionnaire. Within the five thematic areas, we selected 12 indicators pertaining to ART access and eight indicators on ART retention (see Table 1).

**Table 1:** Selected ART access and retention indicators

Policies	Themes in the conceptual framework	Indicators
ART access	Service access and coverage	Proportion of facilities providing ART at no cost
		Proportion of facilities allowing clinical officers and nurses or midwives to initiate ART
	Quality of care	Proportion of facilities providing ART training and refresher courses regularly to health care workers
		Proportion of facilities conducting supervisory visits regularly
		Median number of nurses providing HIV care
		Proportion of facilities with reliable stock levels of ART in the past year
		Proportion of facilities with reliable stock levels of isoniazid preventive therapy (IPT) in the past year
	Medical management	Proportion of facilities requiring no laboratory tests before ART initiation
		Proportion of facilities using WHO ART eligibility threshold as the standard for initiating ART
		Proportion of facilities providing IPT to ART patients
		Proportion of facilities conducting tuberculosis (TB) screening at every visit
		Proportion of facilities providing WHO recommended first-line ART regimen
Retention in care	Coordination of care and patient tracking	Proportion of facilities integrating TB and HIV services
		Proportion of facilities providing three month ART refills
		Proportion of facilities conducting pill count in every visit
	Support to people living with HIV	Proportion of facilities conducting home visits or making calls or sending text messages to patients with suboptimal adherence
		Proportion of facilities conducting home visit or making calls or sending text messages to patients who have missed their visits
		Proportion of facilities encouraging ART patients to join a peer support group
		Proportion of facilities encouraging ART patients to enrol for home based care
		Proportion of facilities providing dietary supplements to undernourished patients

### Data management and analysis

Data from Nairobi, Karonga, Agincourt, Umkhanyakude, Masaka, Rakai, Kisesa and Ifakara were entered into an MS SQL Server (Microsoft Corp) database in Nairobi. Data from Kisumu and Manicaland, were collected on tablets using software developed using the Open Data Kit (ODK) platform (https://opendatakit.org/), and merged with data from the other institutions. Cleaning, merging and analysis of the datasets was carried out using Stata 14 [11].

Descriptive statistics were used to compare selected indicators in each country across the two rounds. The results are presented as the percentage of facilities that reported adherence to each of the indicators in round 1 and 2. Median numbers (with interquartile range (IQR)) were obtained for continuous variables.

## Results

### National-level policy adoption

Seven policy documents were published between July 2013 and July 2015. We identified one policy document from Kenya [12], Malawi [13], Tanzania [14], Uganda [15], Zimbabwe [16] and two from South Africa [17, 18]. All countries had some explicit policies that aimed to increase ART access in line with the WHO 2013 guidelines on ART access and retention in care (see Additional file 1). Kenya, Malawi, South Africa, Tanzania, Uganda and Zimbabwe had adopted policies on decentralisation and nurse-led ART initiation prior to the WHO recommendations. However, no national policy mentioned that lay cadres could distribute ART in the community. Only Malawi’s policies indicated that a three-month supply of ART should be given to clinically stable patients.

### Policy implementation

In total, 145 facilities were included in the analysis, with data collected in both round 1 (2013 to 2015) and round 2 (2015 to 2016). The time between round 1 and round 2 averaged 20.9 months (range 16–25 months). Nineteen facilities were excluded (four from Kenya, five from South Africa, two from Tanzania, and eight from Uganda) because they were not surveyed in both rounds.

### Health facility characteristics

The majority of facilities were either small health centres or dispensaries (75% in round 1 and 82% round 2) and 81% were government owned in both rounds (Table 2). All facilities had a median of three full-time nurses or midwives (IQR: 1.0–5.0 in round 1 and IQR: 2.0–5.5 in round 2) providing HIV services. The proportion of facilities having doctors or clinical officers providing services was 36% in round 1 and 46% in round 2. In round 1, Kenya had the highest median number of weekly ART patients seen per health care worker (11 (IQR: 5–17)) and Malawi had the lowest (1 (IQR: 0–1)). In round 2, South Africa had the highest median number of weekly ART patients seen per health care worker (21 (IQR: 11–44) and again Malawi had the lowest (0 (IQR: 0–2)). In most facilities the caseloads were <50 ART patients/nurse or clinician per week, with only one facility in South Africa in round 2 reporting caseloads of >100 ART patients/nurse or clinician per week.

**Table 2:** Health facility characteristics

	Kenya		Malawi		South Africa		Tanzania		Uganda		Zimbabwe	
	Round 1	Round 2	Round 1	Round 2	Round 1	Round 2	Round 1	Round 2	Round 1	Round 2	Round 1	Round 2
Number of facilities	*N* = 42	N = 42	*N* = 6	N = 6	*N* = 23	N = 23	*N* = 19	N = 19	N = 19	N = 19	*N* = 36	N = 36
Type of facility												
Hospital/large health centres (n(%))	8 (19.0)	7 (16.7)	3 (50.0)	5 (83.3)	0 (0.0)	0 (0.0)	11 (57.9)	10 (52.6)	3 (15.8)	3 (15.8)	11 (30.6)	1 (2.9)
Sub-district hospitals/ health centres/dispensaries (n(%))	34 (81.0)	35 (83.3)	3 (50.0)	1 (16.7)	23 (100.0)	23 (100.0)	8 (42.1)	9 (47.4)	16 (84.2)	16 (84.2)	25 (69.4)	34 (97.1)
Managing authority												
Government (n(%))	34 (81.1)	34 (81.1)	3 (50.0)	3 (50.0)	0 (0.0)	0 (0.0)	16 (84.2)	17 (89.5)	14 (73.7)	14 (73.7)	27 (75.0)	26 (72.2)
Faith-based, NGO, private (n(%))	8 (19.0)	8 (19.0)	3 (50.0)	3 (50.0)	23 (100.0)	23 (100.0)	3 (15.8)	2 (10.5)	5 (26.3)	5 (26.3)	9 (25.0)	10 (27.8)
Median number of nurses/midwives providing HIV services [IQR]	2.8 (2.0–4.0)	3 (2.0–5.0)	1.5 (1.0–4.0)	2 (2.0–3.0)	5 (3.0–6.0)	6 (4.0–7.0)	1 (1.0–6.0)	3.5 (1.0–5.5)	3.5 (1.0–6.0)	2 (2.0–5.0)	1 (1.0–3.0)	1 (0.0–3.0)
Median number of clinicians (doctors and clinical officers) [IQR]	1 (1.0–2.0)	1 (1.0–2.0)	0.5 (0.0–2.0)	1.5 (1.0–3.0)	0 (0.0–0.5)	0 (0.0–0.5)	2 (0.5–2.0)	1 (1.0–5.0)	2 (1.0–3.0)	2 (1.0–3.0)	0 (0.0–0.0)	0 (0.0–0.0)
Median number of weekly ART clients/HIV staff (nurse and clinicians) [IQR]	11 (5–17)	11 (4–18)	1 (0–1)	0 (0–2)	2 (1–12)	21 (11–44)	6 (4–10)	9 (4–19)	11 (6–16)	9 (1–15)	4 (2–15)	6 (2–12)
Median number of ART clients per week (including those starting or coming for a follow-up visit) [IQR]	40 (23–75)	52 (25–92)	3 (2–3)	3 (1–17)	10 (7–43)	151 (70–245)	34 (14–42)	63 (31–216)	49 (24–64)	26 (5–73)	11 (2–30)	11 (7–26)

### Service access and coverage

Across all countries 97% of the facilities in round 1 and 96% in round 2 were providing ART free of charge to all patients. In Malawi, all facilities in both rounds had nurses initiating ART. In other sites, the proportion of facilities with nurses initiating ART increased between round 1 and 2 from 76% to 93% in Kenya, and 67 to 100% in Zimbabwe, with smaller changes in Tanzania (60 to 80%), Uganda (79 to 83%) and South Africa (96 and 96%).

### Quality of care

All facilities in South Africa and Tanzania (in both rounds) reported that they had an HIV treatment and prevention guideline (for any year) (Table 3). The proportion of facilities that had HIV treatment and prevention guidelines increased in Kenya (87 to 100%), and Zimbabwe (40 to 80%), with smaller changes in Malawi (60 to 80%) and Uganda (84 to 100%).

**Table 3:** Quality of care indicators

	Kenya		Malawi		South Africa		Tanzania		Uganda		Zimbabwe	
	Round 1	Round 2	Round 1	Round 2	Round 1	Round 2	Round 1	Round 2	Round 1	Round 2	Round 1	Round 2
Number of facilities	*N* = 38	*N* = 40	*N* = 5	N = 5	N = 23	N = 23	*N* = 15	N = 15	N = 19	*N* = 18	N = 15	*N* = 35
Availability of ART guidelines in the facility (n(%))	33 (86.8)	40 (100.0)	3 (60.0)	4 (80.0)	23 (100.0)	23 (100.0)	15 (100.0)	15 (100.0)	16 (84.2)	18 (100.0)	6 (40.0)	28 (80.0)
Supervisory visits conducted at least once a year (n(%))	36 (94.7)	39 (97.5)	4 (80.0)	5 (100.0)	21 (91.3)	23 (100.0)	14 (93.3)	14 (93.3)	18 (94.7)	18 (100.0)	15 (100.0)	33 (94.3)
First-line ART is in stock (n(%))	31 (81.6)	37 (92.5)	4 (80.0)	5 (100.0)	20 (87.0)	20 (87.0)	10 (66.7)	10 (66.7)	16 (84.2)	15 (83.3)	14 (93.3)	35 (100.0)
ART regimens containing TDF with EFV is in stock (n(%))	4 (10.5)	29 (72.5)	5 (100.0)	5 (100.0)	21 (91.3)	23 (100.0)	2 (13.3)	11 (73.3)	12 (63.2)	17 (94.4)	4 (26.7)	33 (94.3)
IPT is in stock (n(%))	22 (57.9)	37 (92.5)	5 (100.0)	4 (80.0)	20 (87.0)	23 (100.0)	2 (13.3)	2 (13.3)	0 (0.0)	2 (11.1)	8 (53.3)	15 (42.9)
CTX is in stock (n(%))	38 (100.0)	40 (100.0)	5 (100.0)	5 (100.0)	21 (91.3)	22 (95.7)	9 (60.0)	6 (40.0)	19 (100.0)	19 (100.0)	33 (91.7)	34 (97.1)

In both rounds, 75% of facilities indicated that at least one staff member had received training on ART. Almost all facilities received supervisory visits at least once a year in both rounds: Malawi (80 to 100%), South Africa (91 to 100%), Uganda (95 to 100%) Kenya (95 to 98%), Zimbabwe (100 to 94%) and Tanzania (93 and 93%).

No facilities in Malawi and Zimbabwe reported stock-outs of first-line ART in round 2 (from 20 and 7% respectively in round 1). In other countries, stock-outs were still apparent with little change seen in the proportion of facilites that experienced first-line ART stock-out in the past year between round 1 and 2 (Kenya: 18 to 8%, Uganda: 16 to 17%, South Africa: (13 and 13%) and Tanzania: (33 and 33%). Similarly, the overall proportion of facilities reporting stock-outs of regimens containing tenofovir (TDF) with efavirenz (EFV) was similar between round 1 and 2 (18% in round 1 and 11% in round 2).

The proportion of facilites that were stocked with isoniazid preventive therapy (IPT) increased from 58 to 93% in Kenya and from 87 to 100% in South Africa, but showed little change in other countries: Uganda (0 to 11%), Malawi (100 to 80%), Zimbabwe (53 to 43%) and Tanzania (13 and 13%). All facilities in Kenya, Malawi, and Uganda had stocked co-trimoxazole (CTX) in round 1 and 2, with similarly high proportions in South Africa (91 to 96%) and Zimbabwe (92 to 97%). Tanzania had the lowest proprotion of facilities stocking CTX (60 to 40%).

### Medical management

**Fig. 2:**
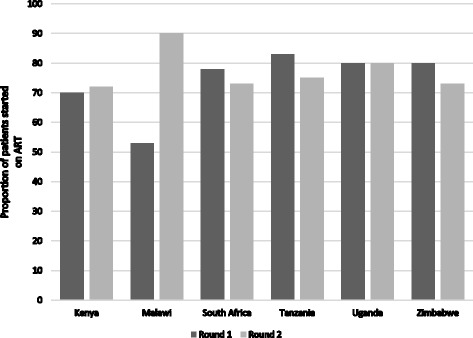
Overall proportion of patients initiating ART within three months of enrolment at clinic, by country for each round

All facilities providing ART services reported a median increase in the patient load from 32 (IQR: 7–60) in round 1 to 36 (IQR: 10–84) in round 2. Taking the denominator as the number of HIV positive patients who enrolled in the clinic, we estimated the proportion who initiated ART within three months. In Malawi, the proportion increased from 53% in round 1 to 90% in round 2, while in all other countries the proportion was unchanged with 70 to 83% of all enrolled HIV positive patients initiating ART within three months (see Fig. 2).

In Tanzania and Malawi, 100% of facilities reported that ART intiation occurred within 2 weeks among patients co-infected with tuberculosis (TB) in round 2 (Tanzania: from 80% in round 1; Malawi: from 80% in round 1). Zimbabwe and Kenya both reported 100% implementation in round 1 with a reduction in round 2 to 91 and 85%, respectively. In Uganda and South Africa, there was little change across rounds the (79 to 89%) and (96 and 96%), respectively.

Positive changes were noted in the proportion of facilities that were starting patients on ART after three or more adherence counselling sessions between round 1 and round 2 in Kenya (13 to 35%), South Africa (48 to 83%), Tanzania (33 to 100%) and Zimbabwe (13 to 100%). Implementation in Uganda remained constant and low (21 to 22%). In both rounds, all facilities in Malawi were starting patients on ART during their first or second visit. Malawi is the only country that did not require a laboratory test before ART initiation in both round 1 and 2. Overall, the proportion of facilities in the other countries providing same-day ART initiation remained low in both round 1 (5%) and 2 (8%).

The percentage of facilities initiating ART patients with a CD4 count of 500 cells/μL or less rose between round 1 and 2 from 0 to 55% in Kenya, 0 to 80% in Malawi, 53 to 91% in South Africa, 0 to 83% in Uganda and 7 to 83% in Zimbabwe. However, this proportion remained the same (6.7%) in Tanzania over the corresponding period as most facilities were initiating patients with a CD4 count of 250 cells/μL or less. Overall, the proportion increased from 12 to 68% between round 1 and 2 in all facilities.

Only Uganda and Zimbabwe had complete information on the CD4 counts of patients at ART initiation. Little change was noted in ART initiation amongst PLHIV with a CD4 count of less than 200 cells/μL between round 1 and round 2 in Uganda (26 to 23%) and Zimbabwe (53 to 57%) nor for ART initiation with CD4 count below 350 cells/μL between round 1 and 2 in Uganda (65 and 66%) and Zimbabwe (90 to 100%).

Nearly all facilities (96% in round 1 and 99% in round 2) were providing CTX prophylaxis to ART patients in both rounds. TB screening of patients at every visit was reportedly carried out by 94% of the health facilities in round 1 and 96% of the health facilities in round 2. The proportion of facilities providing IPT between round 1 and 2 increased from 76 to 95% in Kenya, but there was little change in South Africa (96 to 100%), Tanzania (13 to 20%) and Malawi (100 to 80%). Overall, the proportion of facilities that provided IPT increased from 58 to 71%.

### First-line ART regimens

**Fig. 3:**
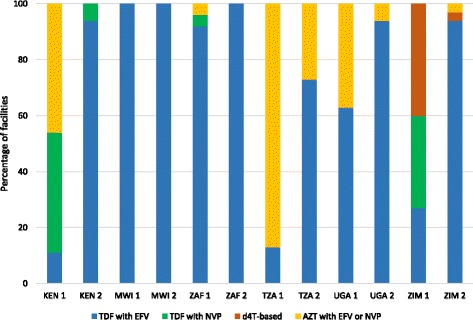
Proportion of health facilities starting patients on different ART regimens during the last three months, by country. *KEN 1-Kenya round 1 *KEN 2-Kenya round 2 *MWI 1-Malawi round 1 *MWI 2-Malawi round 2 *ZAF 1-South Africa round 1 *ZAF 2-South Africa round 2 *TZA 1-Tanzania round 1 *TZA 2-Tanzania round 2 *UGA 1-Uganda round 1 *UGA 2-Uganda round 2 *ZIM 1-Zimbabwe round 1 *ZIM 2-Zimbabwe round 2

The proportion of facilities that prescribed the first-line ART regimen (TDF with EFV) recommended in the 2013 WHO guidelines rose between round 1 and 2 in all countries (Fig. 3). This was universally implemented in Malawi in both rounds. In round 1, six facilities in Zimbabwe were still prescribing d4T-based regimens. However, this dropped to one facility in round 2.

### Retention in care

#### Coordination of care and patient tracking

Integration of HIV and TB services was implemented in all facilities in South Africa and Zimbabwe in both rounds. There were no signficant changes in the proportion of facilites that integrated HIV and TB services between round 1 and 2 in all other countries, with all except Malawi reporting near 90% implementation (Uganda: 78 to 89%, Kenya: 100 to 95%, Tanzania: 93 and 93% and Malawi: 60 and 60%).

There was an overall increase in the proportion of facilities that provided two-month supplies of ART (30 to 49%). However, the proportion remained similarly low in both round 1 (43%) and round 2 (38%) for three-month supplies of ART. Over 90% of all facilities in both rounds (95 to 93%) reportedly allowed ART pick-ups by a designated person. No facility allowed ART patients to pick-up ART supplies in the community.

### Support to people living with HIV

Table 4 shows that implementation of additional strategies to support PLHIV and improve retention were high in both rounds with few changes noted across all countries (conducting home visits and/or making phone calls to patients who missed their clinic visit within two weeks (89 to 96%), establishment of peer support groups (84 to 88%), routine pill counts (74 and 74%). Similarly, high levels of implementation were noted for the provision of home-based care (94 to 87%), and nutritional supplements and food packages to malnourished patients (78 to 77%) in both round 1 and 2.

**Table 4:** Selected retention indicators

Indicators	Kenya		Malawi		South Africa		Tanzania		Uganda		Zimbabwe	
	Round 1	Round 2	Round 1	Round 2	Round 1	Round 2	Round 1	Round 2	Round 1	Round 2	Round 1	Round 2
Number of facilities	N = 38	N = 40	N = 5	N = 5	N = 23	N = 23	N = 15	N = 15	N = 19	N = 18	N = 15	N = 35
Facilities that conducted pill count in every visit (n (%))	30 (78.9)	35 (87.5)	5 (100.0)	5 (100.0)	12 (52.2)	7 (30.4)	10 (66.7)	7 (46.7)	15 (78.9)	12 (66.7)	13 (92.9)	35 (100)
Facilities with missing data (n)											1	
Facilities that conducted home visits or made calls or sent text messages to patients with suboptimal adherence (n (%))	12 (31.6)	19 (47.5)	0 (0.0)	1 (20.0)	1 (4.3)	10 (43.5)	0 (0.0)	2 (13.3)	9 (47.4)	8 (44.4)	4 (26.7)	12 (34.3)
Facilities that conducted home visits or made calls or sent text messages to patients who had missed their visits (n (%))	36 (94.7)	40 (100.0)	4 (80.0)	5 (100.0)	23 (100.0)	23 (100.0)	9 (60.0)	10 (66.7)	18 (94.7)	18 (100.0)	12 (80.0)	35 (100.0)
Facilities that encouraged ART patients to join a peer support group (n (%))	38 (100.0)	36 (90.0)	5 (100.0)	5 (100.0)	13 (92.9)	20 (87.0)	13 (86.7)	10 (66.7)	15 (83.3)	18 (100.0)	13 (86.7)	30 (85.7)
Facilities with missing data (n)					9				1			
Facilities that provided nutritional supplements to those undernourished (n (%))	38 (100.0)	38 (95.00	5 (100.0)	5 (100.0)	23 (100.0)	23 (100.0)	3 (21.0)	3 (20.0)	9 (47.4)	9 (50.0)	12 (80.0)	27 (77.1)
Facilities with missing data (n)							1					
Facilities that encouraged ART patients to enrol for home based care (n (%))	35 (94.6)	29 (72.5)	5 (100.0)	5 (100.0)	22 (100.0)	21 (91.3)	13 (100.0)	13 (100.0)	16 (84.2)	17 (94.4)	15 (100.0)	32 (91.4)
Facilities with missing data (n)	1				1							

## Discussion

This multi-country study has shown a rapid adoption of the 2013 WHO HIV guidelines and significant improvements in the implementation of policies to improve access to ART and retention in care across six countries in SSA by 2017. Our sequential cross-sectional survey rounds between 2013/2015 and 2015/2016 suggest that improvements in ART access have been accompanied by the widespread distribution of suitable guidelines to the facility level, decentralisation of services, high levels of supervisory visits, provision of ART services free of charge, and initiation of ART at higher CD4 counts. Additionally, the health facilities included in the study were increasingly practicing nurse-led ART initiation. This task-shifting approach has been shown to increase both efficiency and effectiveness of ART service delivery [19, 20]. Similar improvements were noted in some strategies to improve retention including improvements to the integration of TB and HIV services, improved provision of sequential adherence counselling sessions, allowing treatment partners to collect medication and continued implementation of support strategies for PLHIV.

Despite this progress, our findings suggest that challenges still remain with implementation of national policies on HIV treatment access and retention in care. For example, although we saw high levels of implementation of the 2013 WHO recommended first-line treatment, a substantial proportion of facilities reported a stock-out in the last three months prior to the survey providing an ongoing cause for concern. Inconsistent stock levels have been shown to have negative consequences on treatment outcomes and patients’ motivations to remain on HIV treatment [21]. Furthermore, recent qualitative work in these HDSS sites suggests that inconsistent stocks of ART prohibits health providers from distributing two- or three- monthly supplies of ART [22]. Longer durations between clinic times are recommended by WHO and Malawi national policies for clinically-stable ART patients and may help to promote retention in care [23]. However, we found that the proportion of facilites providing three-month supplies of ART remained low in both round 1 (43%) and round 2 (38%). Additional efforts are needed to enable facilities to reduce the frequency of clinic visits, particularly as countries will see more “healthy” patients after the implementation of universal test and treat policies. Spacing clinic visits for clinically stable patients has been theorised to free up more time for patients who need more support [23].

No facility was able to provide ART through community outreach, despite policies allowing this in several countries [8]. Additional research is needed to better understand the reasons behind the slow adoption of this strategy. A study in South Africa reported that trained health care workers did not consider such a delivery mechanism as a viable option, believing that the delivery of ART must be done from clinics [24]. Given that current evidence suggests better outcomes among patients utilising this model for adherence support and ART refills [25, 26] and in light of the growing numbers of patient initiating ART as countries adopt 2015 WHO guidelines, countries must develop their own models of differentiated care to meet the local needs.

The facilities in Tanzania showed the least change in the implementation of policies to expand access to and retention on ART between the two survey rounds. Furthermore, we noted that a low percentage of patients initiated ART at higher CD4 counts in Tanzanian facilities despite national adoption of the 2013 WHO guidelines in Tanzania by 2015. It is possible that the frequency with which the WHO treatment guidelines are updated could act as a deterrent for the early uptake and implementation of 2013 WHO guidelines, particularly since roll out strategies take time and resources to implement. Furthermore, in resource-limited settings, the reliability of drug availability remains a challenge, and at the time of the second round, funding negotiations between the Tanzanian government and the Global Fund to Fight AIDS, Tuberculosis and Malaria were still ongoing [27]. Other factors such as stigma, younger age, gender norms [28, 29] and unequal distribution of HIV treatment facilities in the country [30] may have also prevented use of ART at higher CD4 counts in the health facilities.

### Study strengths and limitations

To our knowledge this is the first study to use two sequential cross-sectional surveys to compare implementation of policies on ART access and retention across six African countries with a generalised HIV epidemic. This study also had limitations that should be considered when interpreting the findings. First, the survey was conducted in health facilities serving HDSS sites in each country, and thus the facilities were not selected to be nationally representative. In three large facilities in Rakai, Masaka and Kisumu, additional support was provided for service delivery which could have positively affected the quality of care in comparison to other facilities which operated under typical national supervision. Second, populations within HDSS sites may be different in terms of their treatment-seeking behaviours than those in the general population [31] which may affect the patient volumes witnessed in this study. Third, the completion of the survey by facility managers may present reporting bias and the results may not be a true reflection of the quality of care offered in that facility. Fourth, although the same facilities were used in both surveys, different personnel within the facility may have responded to the survey questionnaire, and any changes in the service may have local reasons (such as personnel changes or transport disruption). Fifth, the analysis on these data were primarily to describe changes, and not to test the significance of any changes.

The conceptual framework that underpinned this study was developed prior to the first facility survey round and was based on a review of literature and policy in circulation up to 2015. Whilst comprehensive at the time, the provision of HIV care and treatment is a rapidly evolving field and it is possible that additional indicators would now be included. Nevertheless this analysis provides a detailed overview of the implementation status of HIV access and retention policies across 145 facilities in six sub-Saharan African countries between 2013 and 2016, a time when considerable changes were taking place at the policy level.

## Conclusion

As the number of people initiating or receiving ART continues to increase, ART service delivery needs to take into account the timeliness of ART initiation following eligibility and enhance programme retention. Overall, there were major improvements in adopting and implementing key policies on increasing ART access and improving retention in most countries. The remaining areas for potential improvement relate to allowing lay cadres to distribute ART in the communities where ART patients live and minimising ART stock-outs. Addressing this area could improve retention among patients now taking ART for longer periods.

## Additional file

Additional file 1: A description of policies for increasing ART access and improving retention in care by country and date of adoption. (DOCX 28 kb)

## Abbreviations

3TC: Lamivudine
ALPHA: Analysing population-based HIV/AIDS data on Africa
ART: Antiretroviral therapy
AZT: Zidovudine
CTX: Co-trimoxazole
d4T: Stavudine
EFV: Efavirenz
HDSS: Health and demographic surveillance site
IPT: Isoniazid preventive therapy
IQR: Interquartile range
NVP: Nevirapine
TB: Tuberculosis
WHO: World Health Organization
