# Supplementary material for: From policy to practice: exploring the implementation of antiretroviral therapy access and retention policies between 2013 and 2016 in six sub-Saharan African countries
Source: BMC Health Serv Res. 2017 Nov 21;17:758. doi: 10.1186/s12913-017-2678-1 (PMC5698969; doi:10.1186/s12913-017-2678-1)
Supplement: Additional file 1: — A description of policies for increasing ART access and improving retention in care by country and date of adoption. (DOCX 28 kb) [file 12913_2017_2678_MOESM1_ESM.docx]

Additional file 1

| **Policy indicator** | **Date of adoption** | | | | | |
| --- | --- | --- | --- | --- | --- | --- |
|  | **Kenya** | **Malawi** | **South Africa** | **Tanzania** | **Uganda** | **Zimbabwe** |
| **ART access** |  |  |  |  |  |  |
| ART should be initiated in patients with a CD4 count of 500 cells/µL and below | June 2014 [1] | April 2014 [2] | December 2014 [3] | May 2015 [4] | December 2013 [5] | December 2013 [6] |
| ART should be initiated in patients co-infected with HIV and hepatitis B virus | June 2014 [1] | April 2014 [2] | December 2014 [3] | May 2015 [4] | December 2013 [5] | December 2013 [6] |
| ART should be initiated in HIV infected pregnant and breastfeeding women | June 2014 [1] | April 2014 [2] | December 2014 [3] | May 2015 [4] | December 2013 [5] | December 2013 [6] |
| ART should be initiated in HIV-infected sero-discordant couples | June 2014 [1] | April 2014 [2] | December 2014 [3] | May 2015 [4] | December 2013 [5] | December 2013 [6] |
| Viral load tests should be used to monitor ART patients | June 2014 [1] | April 2014 [2] | December 2014 [3] | May 2015 [4] | December 2013 [5] | December 2013 [6] |
| Recommended first line ART regimen is TDF + FTC (or 3TC) + EFV | June 2014 [1] | April 2014 [2] | December 2014 [3] | May 2015 [4] | December 2013 [5] | December 2013 [6] |
| Nurses or midwives can initiate and maintain patients on first line ART | December  2005 [7] | April 2008 [8] | April 2010 [9] | April 2012 [10] | May 2003 [11] | May 2010 [12] |
| **Retention in care** |  |  |  |  |  |  |
| SMS reminders messages should be used to improve ART adherence | June 2014 [1] | April 2014 [2] | December 2014 [3] | Not in policy | December 2013 [5] | December 2013 [6] |
| Implement strategies (e.g. appointment system) to reduce waiting times at the clinic | Not in policy | Not in policy | Not in policy | Not in policy | Not in policy | Not in policy |
| ART maintenance can be done in the community | Not in policy | Not in policy | Not in policy | Not in policy | Not in policy | Not in policy |
| Community health workers can dispense ART | Not in policy | Not in policy | Not in policy | Not in policy | Not in policy | Not in policy |

*Tenofovir (TDF)) + emtricitabine (FTC) (or lamivudine (3TC)) + efavirenz (EFV) **Implemented before 2013 WHO recommendations

**References**

1. Ministry of Health Kenya. Guidelines on use of antiretroviral drugs for treating and preventing HIV infection: A rapid advice. National AIDS and STI Control Program, 2014.

2. Ministry of Health Malawi. Malawi Guidelines for Clinical Management of HIV in Children and Adults. National AIDS Commission, 2014.

3. Meintjes G, Black J, Conradie F, Cox V, Dlamini S, Fabian J, et al. Adult antiretroviral therapy guidelines 2014. S Afr J HIV Med. 2014;15(4):121-43.

4. Ministry of Health and Social Welfare Tanzania. National Guidelines for the management of HIV and AIDS. National AIDS Control Programme, 2015.

5. Ministry of Health Uganda. Addendum to the national antiretroviral treatment guidelines. 2013.

6. Ministry of Health and Child Care Zimbabwe. Guidelines for antiretroviral therapy for the prevention and treatment of HIV in Zimbabwe. National Medicine and Therapeutics Policy Advisory Committee and AIDS and TB Directorate, 2013.

7. Ministry of Health Kenya. Guidelines for antiretroviral drug therapy in Kenya. National AIDS and STI Control Program, 2005.

8. Ministry of Health Malawi. Guidelines for the use of antiretroviral therapy in Malawi. National AIDS Commission, 2008.

9. Ministry of Health South Africa. The South African antiretroviral treatment guidelines 2010. National Department of Health, 2010.

10. Ministry of Health and Social Welfare Tanzania. National guidelines for the management of HIV and AIDS. National AIDS control programme, 2012.

11. World Health Organization. Scaling up antiretroviral therapy: experience in Uganda: case study. World Health Organisation, 2003.

12. Ministry of Health and Child Care Zimbabwe. Guidelines for antiretroviral therapy in Zimbabwe. National Drug and Therapeutics Policy Advisory Committee and AIDS and TB Unit, 2010.
